# NRF2-dependent suppression of selenoprotein P expression promotes intracellular selenium metabolic remodeling and upregulation of antioxidant selenoproteins in hepatocellular carcinoma

**DOI:** 10.1016/j.redox.2025.103821

**Published:** 2025-08-13

**Authors:** Kotoko Arisawa, Moeka Natori, Tetta Hiranuma, Misaki Shimizu, Yuto Yamazaki, Yasuhiro Miki, Takashi Toyama, Yoshiro Saito

**Affiliations:** a Laboratory of Molecular Biology and Metabolism, Graduate School of Pharmaceutical Sciences, Tohoku University, 6-3 Aoba, Aramaki, Aoba-ku, Sendai, Miyagi, 980-8578, Japan; b Department of Pathology, Tohoku University Hospital, 2-1 Seiryo-machi, Aoba-ku, Sendai, Miyagi, 980-8575, Japan; c Department of Anatomic Pathology, Tohoku University Graduate School of Medicine, 2-1 Seiryo-machi, Aoba-ku, Sendai, Miyagi, 980-8575, Japan

## Abstract

Selenium-containing antioxidant enzymes such as glutathione peroxidase 4 (GPx4) and thioredoxin reductase 1 (TrxR1, encoded by *TXNRD1*) have emerged as therapeutic targets in hepatocellular carcinoma (HCC), a highly treatment-resistant cancer. Hepatocytes play a central role in selenium metabolism by synthesizing and secreting selenoprotein P (SeP, encoded by *SELENOP*), the major selenium containing protein in plasma, which supplies selenium to peripheral tissues. Although decreased circulating SeP levels have been associated with HCC progression and poor prognosis, the underlying mechanisms remain unclear.

In this study, we reanalyzed publicly available single-cell RNA sequence data of HCC tumors and identified a distinct tumor cell cluster characterized by reduced *SELENOP* expression, enhanced *GPX4* and *TXNRD1* expression, and activation of NRF2 signaling. In HepG2 cells, pharmacological and genetic activation of NRF2 suppressed SeP expression, elevated TrxR1 levels, and promoted intracellular selenium accumulation. Consistently, SeP knockout (KO) cells exhibited increased intracellular selenium, upregulation of GPx1 and GPx4, and resistance to ferroptosis. Similarly, under selenium-deficient dietary conditions, SeP KO mice showed elevated hepatic selenium and GPx1 expression compared to wild-type controls.

These findings uncover a novel NRF2-mediated selenium metabolic remodeling mechanism in HCC, in which SeP suppression promotes intracellular selenium retention and selective upregulation of antioxidant selenoproteins. This redox adaptation contributes to ferroptosis resistance and may represent a potential therapeutic axis in liver cancer.

## Highlights

• Single-cell RNA-seq reveals a tumor cluster with low *SELENOP* and high NRF2 activity in HCC.
• NRF2 activation suppresses SeP expression and increases intracellular selenium levels in HepG2 cells.
• SeP knockout enhances antioxidant selenoproteins and protects cells from ferroptosis in vitro.
• SeP-deficient mice accumulate more liver selenium and GPx1 on low-selenium diets.
• The NRF2–SeP axis remodels selenium metabolism and redox balance in liver cancer.

## Graphical abstract

**Figure:**
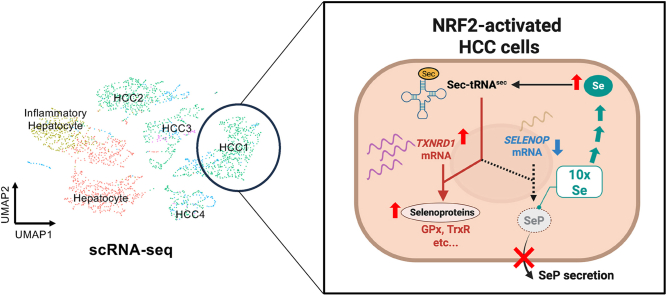

## 1 Introduction

Hepatocellular carcinoma (HCC) is one of the most serious cancers worldwide. As of 2022, it ranks as the sixth most commonly diagnosed cancer and remains a leading cause of cancer-related death [1]. HCC accounts for approximately 75–85 % of primary liver cancers, with chronic infection by hepatitis B or C viruses being the major risk factors [2,3]. Molecular targeted therapies, such as sorafenib and lenvatinib, are used in HCC treatment; however, drug resistance and treatment failure remain major clinical challenges [[4], [5], [6]].

Cancer cells acquire the ability to proliferate under hostile conditions, including hypoxia and elevated oxidative stress, which is largely supported by enhanced antioxidant system [7]. Among these antioxidant pathways, the KEAP1–NRF2 axis serves as a central regulator [8,9]. Constitutive activation of NRF2 has been reported in various cancer types, including HCC [10,11]. Under normal conditions, NRF2 is sequestered in the cytoplasm by KEAP1 and continuously targeted for proteasomal degradation. Upon oxidative or electrophilic stress, KEAP1 undergoes conformational modification, which inhibits NRF2 ubiquitination. As a result, newly synthesized NRF2 accumulates and translocates into the nucleus to activate target gene expression [12]. In HCC and other cancers, mutations or abnormal expression of KEAP1 or NRF2 lead to sustained activation of this pathway, contributing to cancer cell survival, proliferation, and resistance to therapy [13,14]. NRF2 activation is particularly associated with acquired resistance to radiation and chemotherapeutic agents and is considered a marker of poor prognosis in several cancers, including HCC [15,16].

In addition to NRF2-mediated mechanisms, selenium-dependent antioxidant systems play an essential role in redox homeostasis. Selenium is incorporated into proteins as selenocysteine (Sec), forming selenoproteins. In humans, 25 selenoproteins have been identified, many of which are involved in detoxifying reactive oxygen species and maintaining redox balance [17].

Representative antioxidant selenoproteins include glutathione peroxidases (GPxs) and thioredoxin reductases (TrxRs, encoded by *TXNRDs*). TrxR1 contains an antioxidant response element (ARE) in its promoter and is directly regulated by NRF2 [18]. Among the GPx family, GPx2 also possesses an ARE and is directly regulated by NRF2 [19]. GPx1 and GPx4, which are key antioxidant enzymes that protect cancer cells from apoptosis and ferroptosis, do not contain canonical AREs and are not considered direct targets of NRF2 [20,21]. Importantly, elevated expression levels of GPx4 and TrxR1 have been reported as poor prognostic indicators in HCC [22,23].

Selenoprotein P (SeP, encoded by *SELENOP*), which contains ten Sec residues, is primarily synthesized in the liver and secreted into the plasma [24]. It functions as a key regulator of selenium metabolism by supplying Sec to peripheral tissues, supporting the systemic expression of other selenoproteins. Animal models have shown that impaired SeP function leads to selenium deficiency and increased oxidative stress sensitivity in several organs, including the brain, kidneys, and testes [25,26]. Recent studies have reported reduced hepatic expression and circulating levels of SeP in various liver diseases, including nonalcoholic steatohepatitis (NASH) and liver cirrhosis [[27], [28], [29]]. Furthermore, patients with HCC exhibit decreased SeP mRNA expression and reduced SeP protein levels in both tumor tissue and circulation [[30], [31], [32], [33]], which are significantly associated with higher tumor grade, reduced overall survival [32], and poor treatment response [33]. These findings suggest that SeP downregulation may serve as both a prognostic biomarker and a predictive marker of treatment response in HCC.

However, the mechanism underlying the association between decreased SeP expression and disease progression in HCC remains unclear. Although reduced SeP levels are generally linked to diminished selenoprotein expression in peripheral tissues, how its downregulation—particularly in hepatocytes, the primary site of SeP production—affects these cells themselves is still poorly understood. Furthermore, the unique metabolic environment of cancer may cause unexpected changes in selenium utilization and redox regulation [34,35]. In this study, we investigated the expression patterns of SeP and other selenoproteins in HCC by reanalyzing publicly available patient-derived datasets from bulk RNA-seq and single-cell RNA-seq. We also assessed the effects of NRF2 activation on selenoprotein expression in cell-based models. Furthermore, we examined the impact of SeP deficiency on intracellular selenium levels and antioxidant capacity both in vitro and in vivo. Through these analyses, we aimed to elucidate novel mechanisms underlying selenium metabolism and redox control in HCC.

## 2 Materials and methods

### 2.1 Materials

DMEM (high glucose) and 0.25 % trypsin-EDTA were purchased from Nacalai Tesque (Kyoto, Japan). Fetal bovine serum (FBS; BCCC5944), (1S,3R)-RSL3 (SML2234), KI696 (SML3618), and Lipofectamine™ RNAiMAX were from Sigma-Aldrich (St. Louis, MO, USA). Penicillin-streptomycin, PBS (163–25265), CBB G-250 (038–17932), and GAPDH antibody (015–25473) were from Wako Pure Chemical Industries (Osaka, Japan). Cell Counting Kit-8 (343–07623), Liperfluo (L248), and DAB (D006) were from Dojindo (Kumamoto, Japan). Erastin (17754) and cumene hydroperoxide (09816-02) were from Cayman Chemical (Ann Arbor, MI, USA) and Nacalai Tesque, respectively. BODIPY™ 581/591C11 (D3861) and the anti-GPx1 antibody for human samples (MA5-14868) were from Invitrogen (Carlsbad, CA, USA). DC Protein Assay Kit (5000116) and thermal cycler (CFX Connect™) were from Bio-Rad (Hercules, CA, USA). PVDF membranes (IPVH304F0) were from Millipore (Burlington, MA, USA).

KR yeast (YN23L57408) was provided by Mitsubishi Corporation Life Sciences (Tokyo, Japan), and a selenium-deficient AIN-93G-based diet containing this yeast was prepared by Oriental Yeast (Chiba, Japan). The histfine kit was from Nichirei (Tokyo, Japan). ISOGEN II was from NIPPON GENE (Tokyo, Japan). PrimeScript RT Reagent Kit was from Takara Bio (Shiga, Japan), and Power SYBR™ Green PCR Master Mix and NanoDrop spectrophotometer were from Thermo Fisher Scientific (Waltham, MA, USA).

Cas9 plasmid (U-005100-120) was from GE Healthcare (Chicago, IL, USA), and tracrRNA (U-002005-05) and DharmaFECT Duo (T-2010-02) were from Horizon Discovery (Cambridge, UK). Anti-GPx1 antibodies (ab22604, ab125066) and Goat Anti-Rat IgG H&L (Biotin; ab6844) were from Abcam (Cambridge, UK). Anti-hSeP monoclonal antibody (Clone BD1) was used as previously described. Anti-TrxR1 (15140S) was from CST (Danvers, MA, USA), and anti-NRF2 (A-10; sc-365949) was from Santa Cruz (Dallas, TX, USA). Peroxidase-conjugated secondary antibodies for mouse (P0447), rat (P0450), and rabbit (P0448) were from DAKO (Nowy Sącz, Poland). The ImmunoStar LD kit was from FUJIFILM Wako (Osaka, Japan), and Luminograph was from ATTO (Tokyo, Japan).

### 2.2 Immunohistochemistry on patient tissue sections

Liver cancer specimens for immunohistochemistry were collected from inpatients with confirmed pathological diagnoses at Tohoku University Hospital in 2019. The collection of human tissues was approved by the Ethics Committee of Tohoku University Hospital (Approval number: 2024-1-471. Patient data were fully anonymized before access, and no individual information was used. Tissue samples, obtained as residuals from routine pathological examinations following clinical surgery, were paraffin-embedded for immunohistochemical analysis.

Paraffin-embedded sections were dewaxed in xylene and rehydrated through a graded ethanol series. Antigen retrieval was performed using a pressure cooker in antigen retrieval solution. The sections were then treated with 0.3 % hydrogen peroxide in methanol to block endogenous peroxidase activity, followed by blocking with histfine kit. Primary antibody (SeP [BD1 lot; 20191112]) was incubated at 4 °C overnight. After washing, sections were incubated with a secondary antibody (Biotin conjugated anti-Rat IgG) for 30 min at room temperature, followed by DAB color development. Sections were counterstained, dehydrated, and mounted with coverslips.

### 2.3 RNA-seq analysis

RNA-seq data of liver hepatocellular carcinoma (LIHC) were obtained from The Cancer Genome Atlas (TCGA) via the Genomic Data Commons (GDC) portal (https://portal.gdc.cancer.gov/projects/TCGA-LIHC). Gene expression quantification data generated using the STAR - Counts workflow were downloaded and processed using the TCGAbiolinks package in R. TPM (transcripts per million) values were calculated from raw counts using gene lengths derived from GENCODE annotations.

To compare the expression levels of selenoprotein genes between tumor and normal liver tissues, we classified samples based on sample type metadata into “Primary Tumor” and “Solid Tissue Normal” groups. For each gene, we first assessed the normality of TPM distributions using the Shapiro–Wilk test. Because most genes did not follow a normal distribution (p < 0.05), statistical comparisons between tumor and normal tissues were primarily performed using the Wilcoxon rank-sum test (unpaired, two-sided). In addition, for matched tumor–normal pairs derived from the same patients, paired t-tests were conducted to evaluate differential expression where appropriate. Analyses were conducted in R (version 4.4.2), and p-values <0.05 were considered statistically significant.

### 2.4 Single-cell RNA-seq data analysis

Single-cell RNA sequencing data were obtained from a publicly available dataset (Mendeley Data, V1, doi: 10.17632/skrx2fz79n.1 [36]), comprising 38,439 cells from six HCC tumor tissues and 45,354 cells from matched adjacent normal tissues. The data were provided in rds format as Seurat objects and analyzed using Seurat version 4.3.0 in R.

Quality control was performed by excluding cells with fewer than 200 or more than 2500 detected features (nFeature_RNA), and cells with more than 5 % mitochondrial gene expression (percent.mt). Normalization was conducted using the LogNormalize method. Highly variable genes were identified using the FindVariableFeatures function (nfeatures = 2000), followed by data scaling with ScaleData.

Principal component analysis (PCA) was performed using RunPCA, and the top 10 principal components were used for downstream analysis. Clustering was performed using FindNeighbors and FindClusters, and the resulting clusters were visualized by Uniform Manifold Approximation and Projection (UMAP) using the RunUMAP function.

Among the 83,793 cells in the dataset, various cell types including immune cells and stromal cells were represented. To focus specifically on parenchymal liver cells, we extracted 1459 hepatocyte-like cells based on canonical marker gene expression (e.g., *ALB*, *APOA1*) and conducted cluster analysis. This subset enabled us to examine tumor-specific heterogeneity in hepatocyte populations, including malignant hepatocyte clusters.

### 2.5 Cell culture

HepG2 and HuH7 cells were obtained from Cell Resource Center for Biomedical Research, Institute of Development, Aging and Cancer Tohoku University (Miyagi, Japan). HepG2 cells were cultured in high glucose DMEM with 10 % fetal bovine serum (FBS), 100 U/mL and 100 μg/mL penicillin–streptomycin in a humidified incubator under the conditions of 37 °C, 5 % CO2, and 95 % ambient air. Selenium concentration in FBS was 8 μg/L, as measured by ICP-MS, resulting in an approximate final concentration of 10 nM in the culture medium [37]. For cell maintenance, HepG2 cells were cultured in a 10 cm dish passaged at a cell density of 10 % or 20 % and cultured to an 80 % subconfluent state. In the present study, HepG2 was seeded 24 h before the experiments.

### 2.6 KEAP1 inhibition by drug treatment

HepG2 cells (1.2 × 10^5 cells/800 μL/well) were seeded in a 12-well plate and cultured for 24 h. Cells were treated with KI696 at concentrations of 2 μM and 10 μM, along with 100 nM sodium selenite, for 48 h. The same volume of DMSO was used as a control.

### 2.7 Transfection of small interfering RNA

Gene-specific siRNAs targeting the *SELENOP* and *KEAP1* were purchased from Thermo Fisher Scientific (MA, USA). The control siRNA used was SIC-001, a Universal Negative siRNA manufactured by Sigma (MO, USA). HepG2 cells (1.5 × 10^5^ cells/800 μL/well) were seeded in a 12-well plate and transfected simultaneously using 10 nM siRNA and 2 μL Lipofectamine™ RNAiMAX Transfection Reagent per 1 mL of medium. After 48 h of transfection, the cells were harvested for subsequent experiments. The sequences of the siRNAs targeting *SELENOP* genes were as follows: *SELENOP* #1 5′-GCAUAUUCCUGUUUAUCAA-3′, *SELENOP* #2 5′-GCAUAUUCCUGUUUAUCAA-3′, and *SELENOP* #3 5′-GCAUACUGCAGGCAUCUAA-3′. The sequences of the siRNAs targeting *KEAP1* were as follows: *KEAP1*#1 5′-AUAAGCAGGAACCAGGCAU-3′, *KEAP1*#2 5′-UCGUAUUUGACCCAGUCGA-3′, and *KEAP1*#3 5′-UGCUGCACGAGGAAGUCGC-3′.

### 2.8 Generation of CRISPR/Cas9-mediated SeP knock-out HepG2 cells

The CRISPR/Cas9 system was employed to knock out the SeP gene in HepG2 cells. The sgRNA sequence targeting SeP was designed using the CRISPR Design Tool from Horizon Discovery Ltd. (Cambridge, UK). The sgRNA sequence used was: GGCTCTCTGTTCCTCCCGAT. HepG2 cells were cultured in DMEM supplemented with 10 % FBS and 1 % penicillin-streptomycin at 37 °C in a humidified atmosphere containing 5 % CO2. Cells were seeded in 6-well plates at a density of 4.0 × 10^5 cells//well and allowed to reach 70–80 % confluency before transfection. In each well, 100 nmol of crRNA, 100 nmol of tracrRNA, 4 μg of Cas9 plasmid, and 12 mg of DharmaFECT Duo Transfection Reagent were mixed in 400 μL of serum-free DMEM. The mixture was incubated at room temperature for 20 min to allow complex formation. Then, 1600 μL of serum-containing DMEM (without penicillin-streptomycin) was added to each well to create the transfection medium. The medium in each well of the 6-well plate containing cells was removed and replaced with 2 mL of the transfection medium per well.

### 2.9 Selection and screening of transfected cells

Forty-eight hours post-transfection, cells were selected with 1 μg/mL puromycin for 2 days to remove non-transfected cells. Surviving cells were used as SeP knockout cells (SeP KO cells) for further analysis. To obtain a cell line with the same genetic sequence, cell cloning was performed. After 48 h of puromycin selection, cells were washed with PBS, incubated with 0.1 % Trypsin-0.02 % EDTA for 2 min, and detached from the 10 cm dish. Cells were counted and adjusted to a concentration of 2 cells/mL, and 100 μL of the cell suspension was seeded into a 96-well plate. Colonies formed in the wells were then transferred to a 24-well plate, and the cells were sequentially scaled up to larger culture plates as they proliferated. Multiple candidate SeP KO clones and wild-type HepG2 cells were cultured for 48 h in medium containing 100 nM Na2SeO3. These cells were harvested, and SeP knockout was confirmed by Western Blotting.

### 2.10 Western blotting

Cellular proteins were extracted using SDS buffer (0.5 mM Tris-HCl, pH 6.8, 10 % glycerol, 2 % SDS) by heating the samples at 95 °C for 5 min. Protein concentrations were determined using a protein assay kit. Equivalent amounts of proteins were separated by 10 % SDS-PAGE and transferred onto PVDF membranes. The membranes were blocked with 0.01 % polyvinyl alcohol, followed by rinsing with TBST (Tris-buffered saline, pH 7.4, containing 0.1 % Tween 20). The following primary antibodies were used for western blotting: rabbit anti-GPx1 polyclonal antibody (for mouse samples), mouse anti-GPx1 monoclonal antibody (for human samples), rabbit anti-GPx4 monoclonal antibody, rat anti-hSeP monoclonal antibody (Clone BD1) [38], rabbit anti-TrxR1 monoclonal antibody, and rabbit anti-NRF2 polyclonal antibody. After washing with TBST, the membranes were incubated with peroxidase-conjugated secondary antibodies (specific for mouse, rat, and rabbit). Protein bands were visualized using the ImmunoStar LD kit and detected using a Luminograph. Total protein loading was assessed by CBB staining, and GAPDH was used as a loading control. Band intensities were quantified using ImageJ software (NIH). For each membrane, the band intensity of each protein was normalized to the highest intensity band on that membrane.

### 2.11 RNA extraction and quantitative PCR (qPCR)

After treatment, the cell culture medium was discarded, cells were washed with PBS, and ISOGEN II, an RNA extraction reagent, was added. RNA was purified following the manufacturer's instructions, concentration was determined by NanoDrop, and reverse transcription was performed using a PrimeScript RT Reagen Kit. The reagent Power SYBR™ Green PCR Master Mix and a thermal cycler were used for qPCR. All data are normalized by each of GAPDH mRNA level. The primer sequences are provided in the Supplementary Table 1.

### 2.12 Selenium concentration measurement

Selenium levels in cells, mouse serum, and liver were quantified using inductively coupled plasma mass spectrometry (ICP-MS). HepG2 cells were cultured in 10 cm dishes until they reached approximately 90 % confluency. The cells were then washed with PBS and detached from the dishes and centrifuged. The cell pellet was resuspended in 120 μL of PBS. For digestion, 100 μL of this cell suspension was mixed with 250 μL of 70 % nitric acid and 1.25 μL of 100 % hydrochloric acid, then digested using the ETHOS EASY microwave sample preparation system (Milestone General, Kanagawa, Japan) at 160 °C for 30 min. After cooling, the digested samples were diluted with ultra-pure water to a final volume of 1.0 mL. The remaining 20 μL of cell suspension was used to measure protein content for normalization. Mouse serum samples (0.1 mL) were similarly digested with 0.5 mL of 70 % nitric acid using the same microwave system, and liver tissue (10 mg) was digested in 0.5 mL of 70 % nitric acid. All digested samples were diluted to a final volume of 2.0 mL with ultra-pure water.

Selenium was quantified by monitoring the isotope ^78^Se as ^78^SeO in reaction cell mode using oxygen gas to minimize polyatomic interferences. Argon gas (99.996 % purity) was used for plasma generation and nebulization. Quantification was performed using the external calibration curve method with multi-element standard solutions. Internal standards (^9^Be, ^89^Y, ^115^In, ^125^Te, and ^209^Bi) were introduced online with the samples to correct for instrument variability.

Instrumental conditions for ICP-MS analysis are summarized in Supplementary Table 2. To ensure the accuracy and reproducibility of selenium measurements, we validated the method using Seronorm™ Trace Elements Serum (L-2, RUO, lot 1801803, SERO AS, Norway). Validation results, including standard curve parameters, precision, and recovery, are presented in Supplementary Table 3.

Daily instrument performance was evaluated using a 1 μg/L solution containing ^7^Li, ^115^In, and ^238^U, following the manufacturer's guidelines.

The limit of detection (LOD) and limit of quantification (LOQ) for selenium were estimated to be 0.01037 ng/mL and 0.003873 ng/mL, respectively, based on three times and ten times the standard deviation of blank measurements.

### 2.13 Animals

The animal study was carried out in accordance with the rules and guidelines for the proper implementation of animal experiments at 10.13039/501100006004Tohoku University and the experimental plan was approved by the Support Center for Laboratory Animal and Gene Researches, 10.13039/501100006004Tohoku University (Approval No. 2019PhA-018). SeP knockout mice were obtained from The Jackson Laboratory (Bar Harbor, ME, USA), originally generated by homologous recombination with genomic DNA cloned from an Sv-129 P1 library, as previously described (Hill et al., 2003). Male and female SeP heterozygous mice were bred to generate SeP knockout (SeP−/−) and wild-type offspring for experiments. Mice were housed in plastic cages, 4 to 6 per cage, under a 12-h light/dark cycle, with free access to food and water. For routine feeding, CE-2 (CLEA Japan, Tokyo, Japan) was used. For experiments involving selenium-deficient diets, a custom AIN-93G-based feed formulated with KR yeast (YN23L57408) was prepared upon request by Oriental Yeast Co., Ltd. (Chiba, Japan) was used. The selenium content of the custom selenium-deficient diet was measured by ICP-MS and confirmed to be 0.003 mg/kg diet. At the time of sacrifice, mice were anesthetized with inhaled isoflurane, and blood was collected via cardiac puncture under anesthesia. After perfusion with saline, the organs were harvested. Blood samples were left at room temperature for over 20 min, then centrifuged at 3000 rpm at 4 °C for 15 min. The serum was collected and stored at −30 °C. The harvested organs were weighed, frozen in liquid nitrogen, and stored at −80 °C.

### 2.14 Cell viability measurement

Wild-type and SeP KO HepG2 cells (2.0 × 10^4^ cells/100 μL/well) were seeded in a 96-well plate and cultured at 37 °C, 5 % CO2, and 95 % ambient air for 24 h. Cells were then treated with 0.5, 1, 3, 5 μM Erastin or 25, 50, 100 nM RSL3. The medium was replaced with a new medium containing FBS and 1/10 volume of Cell Counting Kit-8, and cells were incubated for 1 h and 30 min. Absorbance 450 nm was measured using a plate reader.

### 2.15 Cell imaging of lipid hydroperoxide

Wild-type and SeP KO HepG2 cells (8.0 × 10^4^ cells/400 μL/well) were seeded in 8-well chambers and cultured for 24 h. Cells were incubated with 20 μM Liperfluo in serum-containing medium for 30 min, followed by replacement with fresh DMEM containing 100 μM cumene hydroperoxide for an additional 30 min. After washing once with PBS and replacing with fresh DMEM, cells were observed under a confocal microscope. In experiments measuring lipid hydroperoxidation in ferroptosis, cells were treated with 3 μM Erastin or 100 nM RSL3 for 24 h, followed by incubation with 20 μM Liperfluo for 30 min. After replacing with fresh DMEM, cells were observed under a confocal microscope. For fluorescence measurement using a plate reader, cells (2.0 × 10^4^/100 μL/well) were seeded in 96-well plates and subjected to the same treatments for fluorescent staining as described previously. After washing the cells with PBS and replacing it with fresh PBS, fluorescence was measured at Ex/Em 485/580 nm.

### 2.16 Statistical analysis

All experiments were repeated independently with similar results at least three times. Data are presented as the mean ± standard deviation (SD), with a sample size of n ≥ 3 for each experiment. Statistical analyses were performed using GraphPad Prism 10 software (GraphPad Software Inc., CA, USA). Welch's *t*-test was used for comparisons between two groups. Dunnett's test or Tukey's test was applied as appropriate. Two-way ANOVA followed by Dunnett's multiple comparison test was used to assess the effects of two independent variables (e.g., cell type and treatment concentration) using wild-type cells as the control. A p-value below 0.05 was considered statistically significant.

## 3 Result

### 3.1 Differential expression of selenoproteins in HCC tissues

**Fig. 1:**
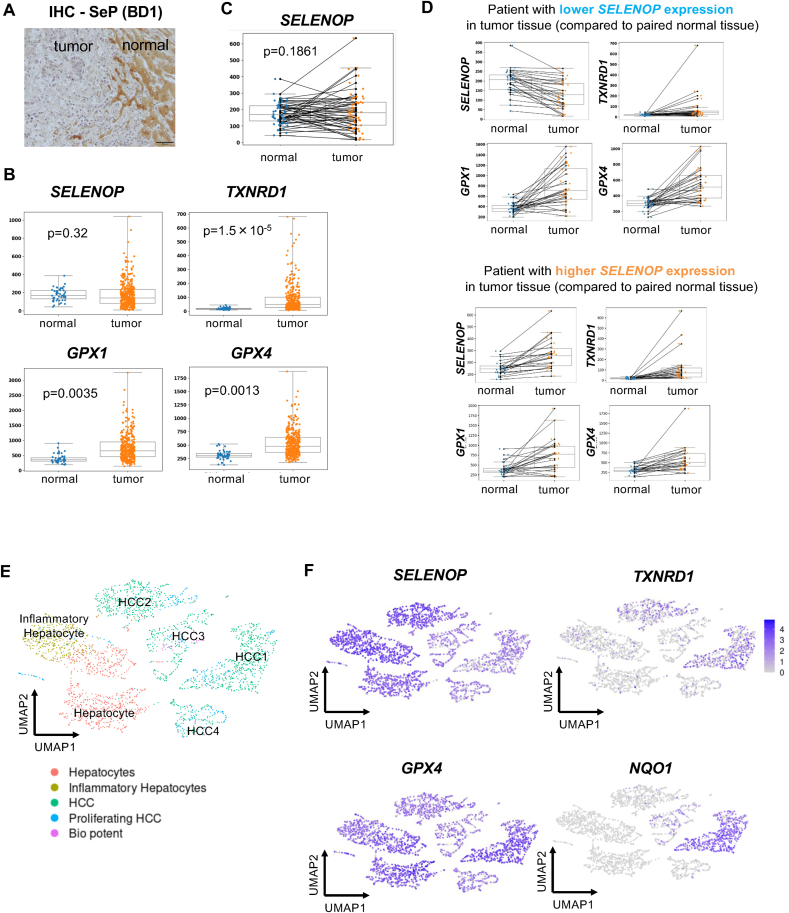
Gene expression analysis of selenoproteins in HCC patients using TCGA and single-cell RNA-seq datasets. (A) Immunohistochemical staining of SeP in human HCC tissues. Representative images show SeP localization in tumor and adjacent normal regions. Scale bar, 50 μm (B) Box plots showing the transcript levels of *SELENOP*, *TXNRD1*, *GPX1*, and *GPX4* in tumor (n = 371) and normal liver tissues (n = 50) from the TCGA-LIHC RNA-seq dataset. Expression values are shown as TPM, and statistical differences were evaluated using the Wilcoxon rank-sum test. **(C)** Paired expression analysis of *SELENOP* in tumor and adjacent normal tissues from 50 HCC patients in the TCGA-LIHC dataset. Statistical significance was assessed using paired t-tests. **(D)** Paired expression analysis of *TXNRD1*, *GPX1*, and *GPX4* in the same patient dataset as in (B). Patients were stratified into two subgroups based on whether *SELENOP* expression was decreased (upper) or increased (lower) in tumor tissues to matched normal tissues. **(E)** UMAP visualization of 1459 hepatocyte-like cells extracted from a publicly available single-cell RNA-seq dataset containing 83,793 cells from six HCC tumor tissues and their matched adjacent normal tissues (Xun et al., 2022). Hepatocyte-like cells were identified using canonical marker gene expression and clustered into six groups: normal hepatocytes, inflammatory hepatocytes, and four tumor-associated clusters (HCC1–4). **(F)** UMAP showing the expression of *SELENOP*, *TXNRD1*, *GPX4*, and *NQO1* across the six hepatocyte clusters identified in (E), based on normalized single-cell RNA-seq counts.

Immunohistochemical (IHC) analysis using our in-house SeP antibody (BD1), which recognizes the N-terminal region [38], showed reduced SeP protein levels in tumor regions compared to adjacent normal regions in liver tissue sections from HCC patients (Fig. 1A). This staining pattern was consistent with previous reports using different SeP antibodies [31]. This protein-level suppression supported the reliability of SeP downregulation in HCC tumors and prompted us to investigate whether similar trends were evident at the transcriptomic level in a larger cohort. Although discrepancies between mRNA and protein levels are well known for selenoproteins, we analyzed publicly available transcriptomic data from a large-scale cohort to assess potential expression patterns.

To investigate the expression profiles of *SELENOP* and other selenoproteins in HCC, we analyzed RNA-seq data from the TCGA-LIHC dataset obtained via GDC portal. Comparison between tumor tissues (n = 371) and normal liver tissues (n = 50) revealed no statistically significant difference in *SELENOP* expression using the Wilcoxon rank-sum test (Fig. 1B). However, *TXNRD1*, *GPX1*, and *GPX4*, which are key antioxidant selenoproteins, showed significantly higher expression in tumors. Notably, increased expression of *GPX4* and *TXNRD1* has been reported as a poor prognostic indicator in HCC. In total, 14 out of the 25 human selenoprotein genes showed significantly higher expression in tumors (p < 0.05; Supplementary Fig. 1 and Supplementary Table 4), while the remaining genes showed no significant change. These results suggest that selenoprotein expression is broadly upregulated at the transcript level in HCC.

To further assess intra-individual differences, we analyzed paired tumor and matched adjacent normal tissue from 50 patients (Fig. 1C). Paired *t*-test analysis confirmed no significant change in *SELENOP* expression, while 21 out of 25 selenoprotein genes showed significantly increased expression in tumor tissues compared to matched normal tissues (Supplementary Table 5). Interestingly, when patients were stratified based on tumor-associated increases or decrease in *SELENOP* expression relative to their own normal tissue, most other selenoproteins were consistently upregulated in tumors in both subgroups (Fig. 1D). These findings suggest that, regardless of inter-patient variability in *SELENOP* expression, the expression of most selenoproteins is broadly elevated in HCC tissues.

To resolve this discrepancy between transcript and protein levels, we hypothesized that *SELENOP* suppression may occur within a specific subpopulation of tumor cells. To test this, we performed single-cell RNA sequencing analysis using a publicly available dataset comprising 38,439 cells from six HCC tumor tissues and 45,354 cells from matched adjacent normal tissues (Xun et al., 2022; Mendeley Data, V1, doi: 10.17632/skrx2fz79n.1). A total of 1459 hepatocytes were extracted based on marker gene expression, and UMAP-based clustering identified two hepatocyte-related clusters (normal hepatocytes and inflammatory hepatocytes) and four tumor-enriched clusters (designated HCC1–4) (Fig. 1E).

Cluster-specific analysis showed markedly reduced *SELENOP* expression in the HCC1 cluster, whereas *TXNRD1* expression was elevated and *GPX4* remained relatively unchanged (Fig. 1F). In contrast, the HCC2 cluster retained *SELENOP* expression levels comparable to those of normal hepatocytes, with moderate upregulation of *TXNRD1* and a slight decrease in *GPX4*. *GPX1* expression was not annotated in this dataset and could not be evaluated. Additionally, the HCC1 cluster exhibited increased expression of *NQO1*, a well-known NRF2 target gene (Fig. 1F). Given that *TXNRD1* is also regulated by NRF2 and contains an ARE in its promoter region, and that NRF2 activators such as sulforaphane have been reported to downregulate *SELENOP* expression [39,40], these findings collectively suggest that the HCC1 cluster is characterized by enhanced NRF2 activity, which may underlie its distinctive selenoprotein expression profile.

### 3.2 NRF2 regulation of selenoprotein expression in HepG2 cells

**Fig. 2:**
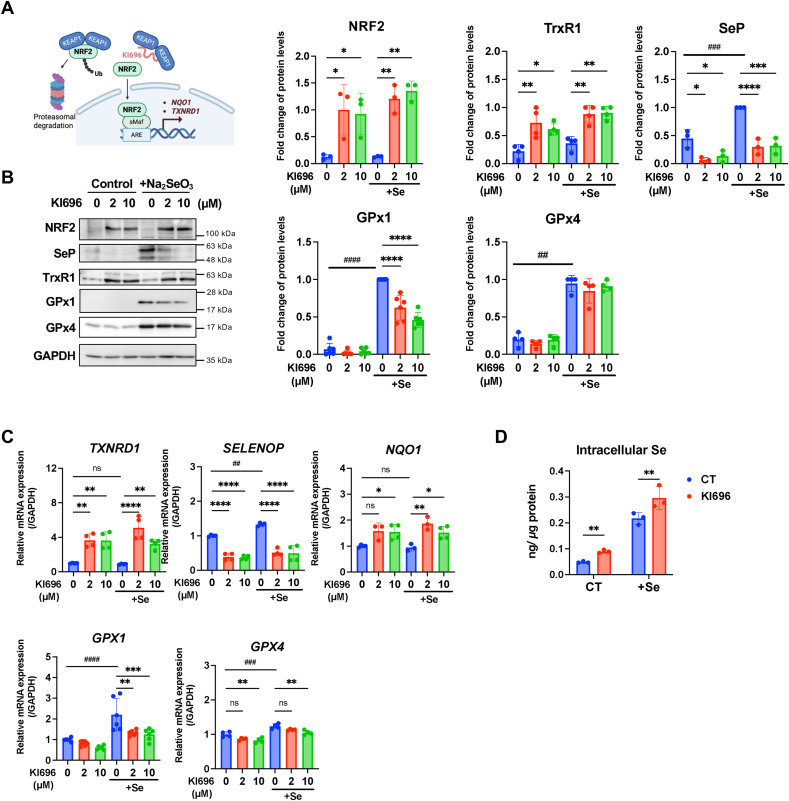
Effects of NRF2 activation on selenoprotein expression in HepG2 cells. (A) Schematic overview of NRF2 activation by KI696. KI696 is a selective inhibitor that targets the Kelch domain of KEAP1 to stabilize and activate NRF2. (B) HepG2 cells were treated with 0, 2, or 10 μM KI696 for 48 h, with or without the addition of 100 nM sodium selenite. Protein expression was analyzed by western blotting and normalized to GAPDH. Band intensities were quantified relative to the highest signal on each membrane. Data are presented as mean ± SD (n = 3–6). Statistical significance: ∗p < 0.05, ∗∗ <0.01, ∗∗∗ <0.001, ∗∗∗∗ <0.0001 vs. 0 μM KI696 within each selenium condition; #p < 0.05, ## < 0.01, ### < 0.001, #### < 0.0001 for Se– 0 μM KI696 vs. Se+ 0 μM KI696 (Tukey's multiple comparison test). (C) mRNA expression levels under the same treatment conditions as in (B), normalized to *GAPDH*. Quantification was performed by qRT-PCR. (D) Intracellular selenium levels were measured by ICP-MS under the same experimental conditions as in (B). Selenium concentrations were normalized to total protein content. Data are presented as mean ± SD. ∗∗p < 0.01 vs. control (CT) within each selenium condition (Šídák's multiple comparison test).

Based on these observations, we hypothesized that SeP and other selenoproteins in HCC are regulated by NRF2. To test this, we examined the effects of NRF2 activation on selenoprotein expression in HepG2 cells. Treatment with KI696, a selective inhibitor of NRF2–KEAP1 binding (Fig. 2A), increased total NRF2 protein levels and upregulated *NQO1* mRNA expression, confirming NRF2 activation (Fig. 2B and C). This activation was accompanied by increased TrxR1 protein levels, consistent with previous reports. Interestingly, under normal culture conditions without selenium supplementation, we found that NRF2 activation led to a marked decrease in SeP protein and mRNA levels, while GPx1 and GPx4 protein and mRNA levels remained largely unchanged (Fig. 2B and C).

Intracellular selenium levels measured by ICP-MS were significantly increased following KI696 treatment, representing approximately a 1.9-fold increase (Fig. 2D). Selenium supplementation led to a marked increase in intracellular selenium relative to control, and the combination of selenium and KI696 further enhanced intracellular selenium levels beyond selenium alone.

Supplementation with sodium selenite increased the expression of several selenoproteins except TrxR1. Under these selenium-replete conditions, KI696 treatment markedly reduced SeP protein levels, while simultaneously enhancing TrxR1 and reducing GPx1 expression (Fig. 2B). At the mRNA level, KI696 increased *TXNRD1* expression and decreased *SELENOP* expression. *GPX1* and *GPX4* mRNA levels were upregulated by selenium supplementation, but KI696 treatment reduced their expression to levels comparable to untreated controls (Fig. 2C).

A similar pattern of mRNA expression was observed upon NRF2 activation via *KEAP1* knockdown using two independent siRNA sequences (Supplementary Fig. 3). Collectively, these results suggest that NRF2 activation may contribute to SeP downregulation and influence intracellular selenium levels and the expression of selenoproteins in hepatocytes.

### 3.3 SeP knockout increases cellular selenoprotein expression and selenium levels in HepG2 cells

**Fig. 3:**
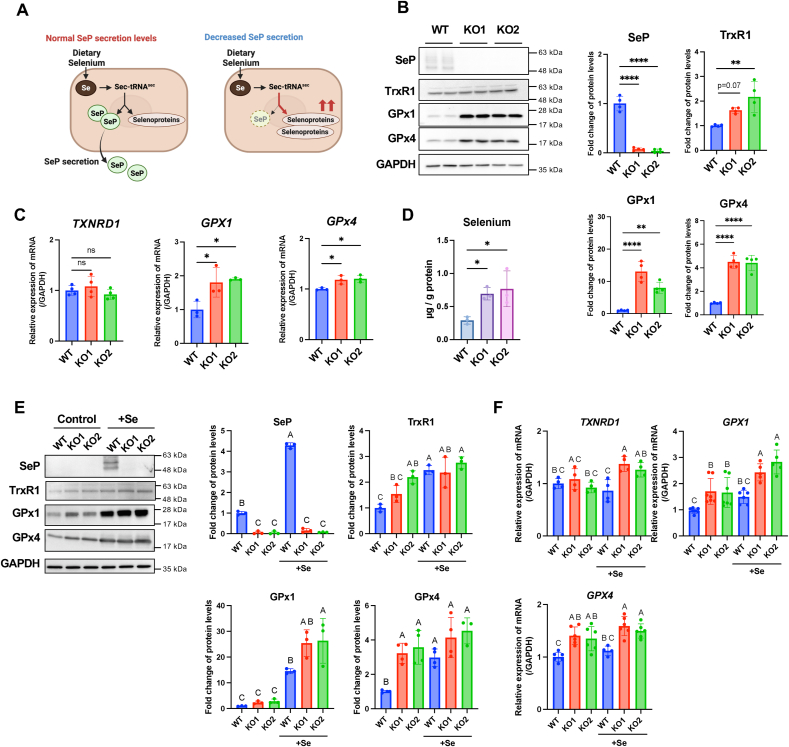
The impact of SeP knockout on cellular selenoproteins expression and selenium levels in HepG2 cells (A) Schematic diagram illustrating the relationship between SeP secretion and cellular selenoprotein expression in hepatocytes. (B) Expression of selenoproteins in SeP KO cells. wild-type (WT) and SeP KO HepG2 cells were cultured in medium without sodium selenite for 24 h, and intracellular selenoprotein expression levels were compared using Western blotting. Data are presented as mean ± SD (n = 3–6). Statistical significance: ns, not significant; ∗p < 0.05, ∗∗ <0.01, ∗∗∗ <0.001, ∗∗∗∗ <0.0001 vs. WT, analyzed by Dunnett's test. (C) mRNA levels of selenoproteins in SeP KO cells under the same treatment conditions as in (B), normalized to GAPDH. Quantification was performed by qRT-PCR. (D) Intracellular selenium levels were measured by ICP-MS. Cells were cultured until ∼90 % confluency, and selenium concentrations were normalized to total protein content. Data are presented as mean ± SD. ∗∗p < 0.01 (Welch's *t*-test). (E) Selenoprotein expression following sodium selenite supplementation. WT and SeP KO cells were treated with 100 nM sodium selenite for 48 h, and protein levels were assessed by Western blotting. Data are shown as mean ± SD (n = 4–6). Groups with different letters (A, B, C) are significantly different (p < 0.05, Tukey's test). (F) mRNA levels of selenoproteins under the same treatment conditions as in normalized to GAPDH. Quantification was performed by qRT-PCR.

Given the increase in intracellular selenium levels following KI696 treatment (Fig. 2D), we investigated the impact of SeP downregulation on selenium homeostasis by generating SeP knockout (SeP KO) HepG2 cells using CRISPR/Cas9. As hepatocytes are the primary source of SeP secretion into extracellular fluids, we examined how blocking SeP synthesis and secretion affects intracellular selenoprotein expression (Fig. 3A).

SeP KO cells showed a marked reduction in SeP protein levels and exhibited significantly increased TrxR1, GPx1, and GPx4 protein levels compared to wild-type (WT) cells (Fig. 3B). qPCR analysis revealed elevated *GPX1* mRNA levels and a modest but statistically significant increase in *GPX4* mRNA in SeP KO cells, while *TXNRD1* mRNA levels remained largely unchanged (Fig. 3C). Given that *GPX1* and *GPX4* translation is tightly regulated by intracellular selenium availability [[41], [42], [43]], these results suggest that increased GPx1 and GPx4 protein levels likely reflect selenium-dependent translational control rather than strong transcriptional induction. ICP-MS analysis demonstrated a 2.3-fold increase in intracellular selenium levels in SeP KO cells (Fig. 3D).

Supplementation with 100 nM sodium selenite increased SeP, TrxR1, GPx1, and GPx4 protein levels in WT cells (Fig. 3E). Upon selenium supplementation, the differences in TrxR1 and GPx4 expression between WT and SeP KO cells were diminished; however, GPx1 levels remained consistently higher in SeP KO cells regardless of selenium treatment. At the mRNA level, *TXNRD1* expression showed minimal change, whereas *GPX1* and *GPX4* remained elevated in SeP KO cells under both selenium-replete and selenium-deficient conditions (Fig. 3F).

These findings suggest that suppression of SeP synthesis and secretion leads to increased intracellular selenium retention, which in turn enhances the expression of multiple selenoproteins.

### 3.4 SeP knockout inhibits lipid hydroperoxide accumulation in HepG2 cells

Given the elevated intracellular selenium levels and increased expression of antioxidant selenoproteins such as GPx1 and GPx4 in SeP KO cells, we hypothesized that the redox balance may be altered in these cells, resulting in enhanced antioxidant capacity. In particular, the upregulation of GPx4, which catalyzes the reduction of lipid hydroperoxides to non-toxic lipid alcohols, led us to examine whether SeP deficiency affects intracellular lipid hydroperoxide levels. To assess this, we used Liperfluo, a fluorescent probe that specifically reacts with lipid hydroperoxides.

**Fig. 4:**
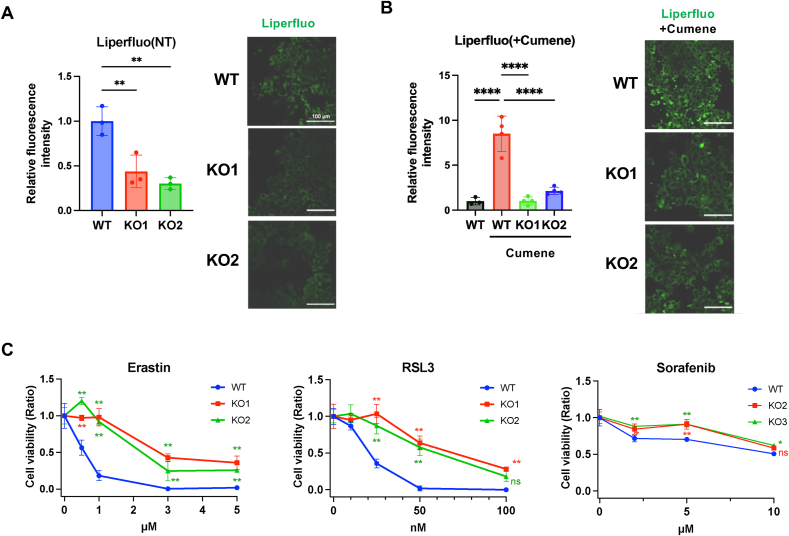
Suppression of lipid hydroperoxide accumulation and ferroptosis in SeP knockout HepG2 Cells (A) Lipid hydroperoxide was assessed using Liperfluo. Cells were incubated with 20 μM Liperfluo, and fluorescence intensity was measured using a plate reader (Ex/Em: 485/580 nm) and fluorescence microscopy. Fluorescence values from the plate reader were normalized to cell viability. Scale bar, 100 μm. Data are presented as the means ± SD (n = 3, biologically independent replicates). Statistical analysis was performed using one-way ANOVA followed by Dunnett's post hoc test. ns, not significant; ∗∗p < 0.01 vs. WT cells. (B) Lipid peroxidation following cumene hydroperoxide exposure. Cells were treated with 50 nM cumene hydroperoxide for 30 min, and lipid hydroperoxide was measured using Liperfluo. Scale bar, 100 μm. Data are presented as mean ± SD. Statistical analysis was performed using one-way ANOVA followed by Dunnett's post hoc test. ns, not significant; ∗∗∗∗p < 0.0001 vs. WT cells treated with cumene hydroperoxide. (C) Sensitivity to ferroptosis inducers. Cells were treated with Erastin (0.5, 1, 3, or 5 μM), RSL3 (10, 25, 50, or 100 nM), or Sorafenib (3, 5, or 10 μM) for 24 h. Cell viability was determined by WST-8 assay. Data are presented as mean ± SD (n = 3). Statistical analysis was performed using a two-way ANOVA followed by Dunnett's post hoc test. ns, not significant; ∗∗p < 0.01 versus WT cells for each reagent.

At baseline, SeP KO cells exhibited significantly lower lipid hydroperoxide levels compared to wild-type (WT) cells (Fig. 4A). To evaluate the ability to eliminate excess lipid hydroperoxides under oxidative challenge, cells were treated with cumene hydroperoxide, an organic peroxide known to induce lipid peroxidation. In WT cells, cumene hydroperoxide treatment led to a marked increase in lipid hydroperoxide accumulation, whereas this response was significantly attenuated in SeP KO cells (Fig. 4B).

Given the suppression of lipid hydroperoxides, we next assessed whether SeP KO cells exhibit altered sensitivity to ferroptosis, a form of regulated cell death triggered by iron-dependent lipid peroxidation. Cells were treated with the ferroptosis inducers erastin or RSL3 for 24 h. The ferroptotic nature of cell death in this model was validated by co-treatment with deferoxamine, an iron chelator, which rescued cell viability (Supplementary Fig. 5). Notably, SeP KO cells exhibited greater resistance to ferroptosis, maintaining significantly higher viability than WT cells at 0.5 μM erastin and 25 nM RSL3 (Fig. 4C). A similar trend was observed with sorafenib, a clinically used multi-kinase inhibitor that has been reported to induce ferroptosis under certain conditions.

Together, these results demonstrate that SeP deficiency confers protection against lipid hydroperoxide accumulation and ferroptosis, likely via enhanced expression of antioxidant selenoproteins. These findings suggest that SeP acts as a modulator of ferroptotic sensitivity by controlling intracellular selenium allocation and selenoprotein expression.

### 3.5 Increased selenoprotein expression and selenium levels in the liver of SeP KO mice

**Fig. 5:**
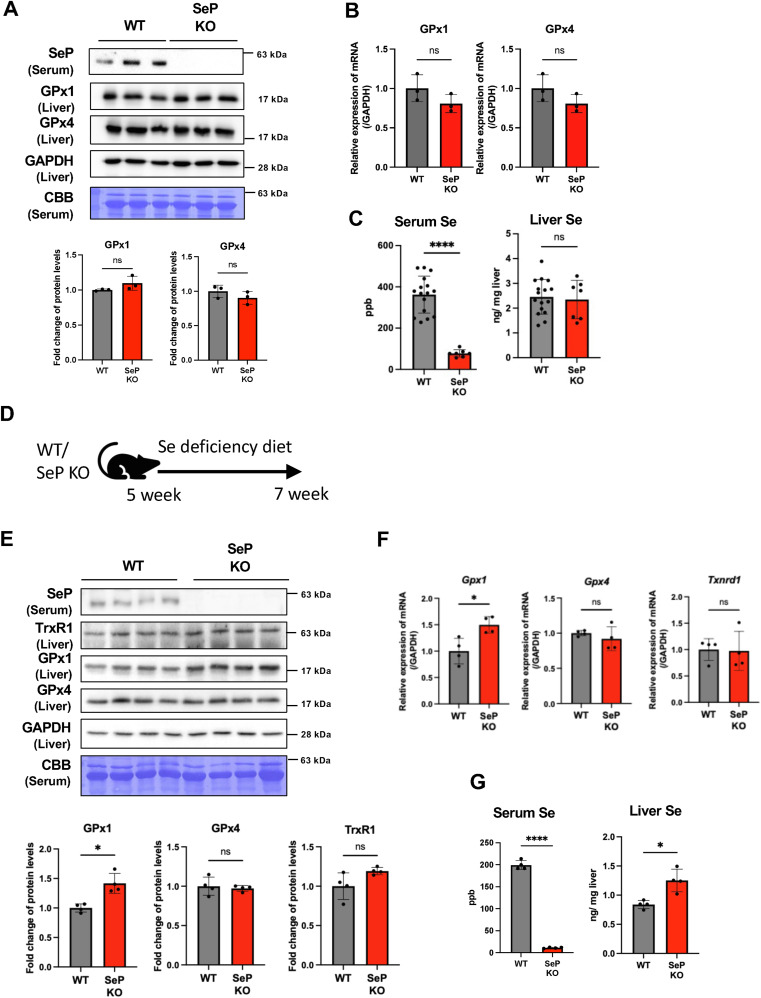
Selenoprotein expression and selenium levels in SeP KO mice under standard and selenium-deficient dietary conditions. (A) Western blot analysis of selenoprotein expression in serum and liver samples from 12-week-old WT and SeP KO mice under standard feeding conditions. (B) mRNA expression levels of selenoproteins in liver samples from WT and SeP KO mice under standard diet conditions. Total RNA was extracted, and transcript levels were measured by RT-qPCR and normalized to GAPDH. (C) Selenium concentrations in serum and liver from WT (n = 16) and SeP KO (n = 7) mice, measured by ICP-MS. (D) Experimental timeline of selenium-deficient diet feeding. WT and SeP KO mice were derived from the same breeding colony. All mice were maintained under standard feeding conditions until 5 weeks of age, after which they were fed a selenium-deficient diet for 14 days (from 5 to 7 weeks of age). (E) Selenoprotein expression in serum and liver of WT and SeP KO mice fed a selenium-deficient diet from 7 to 9 weeks of age. Protein levels were assessed by Western blotting. Quantification was performed from independent membranes, normalized to GAPDH (liver) or CBB staining (serum). (F) mRNA expression levels of selenoproteins in liver samples from selenium-deficient diet-fed WT and SeP KO mice. (G) Selenium concentrations in serum and liver samples from selenium-deficient diet-fed mice. For (A)–(G), data are presented as means ± SD. n = 3–4 (except for panel C, see above). Statistical analysis was performed using Welch's *t*-test. ns: not significant; ∗p < 0.05; ∗∗p < 0.01; ∗∗∗p < 0.001 vs. WT. All blots were performed using independent membranes, with equal amounts of protein loaded in each lane.

To investigate the role of SeP in regulating selenium homeostasis in vivo, we analyzed selenoprotein expression and hepatic selenium distribution in SeP KO mice. Mice were maintained on a standard CE-2 diet containing 0.34 mg Se/kg, which exceeds the recommended dietary allowance of 0.1 mg Se/kg [25]. Under these selenium-replete conditions, hepatic GPx1 and GPx4 protein expression levels in SeP KO mice were comparable to those in wild-type (WT) mice (Fig. 5A and B). While serum selenium levels were significantly reduced in SeP KO mice—consistent with the absence of circulating SeP—hepatic selenium content remained unchanged (Fig. 5C).

To further examine the effects of selenium restriction, mice were fed a selenium-deficient diet (0.003 mg Se/kg) for two weeks, and hepatic selenoprotein expression was assessed (Fig. 5D). In WT mice, selenium deficiency resulted in decreased serum SeP, reduced selenium concentrations, and lowered hepatic GPx1 expression (Supplementary Fig. 6). In contrast, SeP KO mice exhibited a significant increase in hepatic GPx1 expression at both the protein and mRNA levels (Fig. 5E and F), whereas GPx4 and TrxR1 expression remained unchanged. Serum selenium concentrations were markedly decreased in selenium-deficient WT mice (from 363 ± 90 ppb to 199 ± 10 ppb) and dropped further in SeP KO mice (from 77.7 ± 18 ppb to 11.1 ± 1.3 ppb) (Fig. 5C and G). Despite the overall selenium restriction, hepatic selenium content was significantly higher in SeP KO mice compared to WT mice under selenium-deficient conditions (Fig. 5G).

Together with our in vitro findings, these results indicate that the loss of SeP-mediated selenium export leads to hepatic selenium retention and compensatory upregulation of specific selenoproteins such as GPx1. These findings suggest a regulatory mechanism by which SeP deficiency shifts selenium utilization toward hepatic retention and increased selenoprotein synthesis.

## 4 Discussion

This study suggests that NRF2 activation suppresses SeP expression and secretion in hepatocytes, leading to intracellular selenium retention and subsequent upregulation of antioxidant selenoproteins such as GPx1, GPx4, and TrxR1. Analysis of TCGA-LIHC bulk RNA-seq data and publicly available single-cell RNA-seq data revealed heterogeneous *SELENOP* expression across HCC tumors, with marked downregulation in NRF2-activated clusters. NRF2 activation in HepG2 cells reproduced this phenotype. SeP knockout in HepG2 cells and mice demonstrated that SeP suppression promote intracellular selenium retention and ferroptosis resistance in hepatocytes. These findings support the notion that SeP functions as a central modulator linking NRF2 signaling, selenium distribution, and redox adaptation in HCC.

Previous studies have reported that patients with HCC exhibit decreased *SELENOP* mRNA levels and lower serum selenium concentrations [[30], [31], [32]]. Recent advances in single-cell transcriptomics have also highlighted the intratumoral heterogeneity of gene expression in HCC [44]. Within a publicly available single-cell transcriptomic dataset of HCC tumors, we identified a tumor cluster (HCC1) with low *SELENOP* expression and high NRF2 activity. This suggests that SeP downregulation may reflect a metabolic adaptation in NRF2-activated tumor subpopulations. Given that NRF2 is frequently activated in HCC and other cancers via KEAP1 mutations or oxidative stress [13,18], such tumor clusters could represent functionally relevant subpopulations in cancer progression.

To investigate the regulatory role of NRF2 on SeP expression, we activated NRF2 in HepG2 cells using both a selective small-molecule inhibitor (KI696) and genetic knockdown of KEAP1. Previous studies have reported that treatment with NRF2 activators such as diethyl maleate, sulforaphane, and cardamonin led to a reduction in *SELENOP* mRNA levels in HepG2 cells [39]. Our earlier work further showed that sulforaphane suppresses SeP protein levels in a manner partially independent of NRF2 [40]. However, because sulforaphane and other electrophiles lack target specificity and may disrupt broader aspects of selenium metabolism [45], in this study, we employed NRF2-specific strategies to more precisely assess the effect of NRF2 activation on SeP and related pathways.

Consistent with our hypothesis, NRF2 activation led to increased expression of canonical targets such as *NQO1* and *TXNRD1*, accompanied by significant suppression of *SELENOP*. Interestingly, while *GPX4* mRNA levels were slightly reduced, its protein expression remained stable. Furthermore, intracellular selenium concentrations increased upon NRF2 activation.

This discrepancy between mRNA and protein levels may be explained by the concept of the “selenoprotein hierarchy”, which describes how sensitivity to selenium availability varies among selenoproteins due to factors such as UGA recoding efficiency and the involvement of cofactors like SBP2 [[46], [47], [48]]. *GPX4* mRNA levels are known to remain relatively stable even under selenium-deficient conditions; however, its translation is tightly regulated by intracellular selenium levels via SECIS element–dependent mechanisms, and is enhanced by selenium supplementation [41,42]. In contrast, GPx1 expression is more sensitive to selenium status at both the transcriptional and translational levels, showing marked suppression under selenium limitation [43]. TrxR1 maintains relatively stable translation efficiency regardless of selenium levels.

Taken together, our findings suggest that while NRF2 activation may suppress *GPX1* and *GPX4* transcriptionally, the concurrent increase in intracellular selenium—potentially due to SeP downregulation—could sustain or enhance its translation. This implies that NRF2-driven changes in selenium distribution, via reduced SeP expression, may indirectly contribute to the maintenance of antioxidant selenoprotein levels in hepatocytes.

Although the precise molecular mechanism by which NRF2 suppresses *SELENOP* mRNA expression remains unclear, it is noteworthy that NRF2—while primarily known as a transcriptional activator—has also been shown to repress gene expression under certain conditions. For instance, a previous study demonstrated that NRF2 can inhibit the transcription of proinflammatory cytokines such as IL-6 and IL-1β by interfering with RNA polymerase II recruitment, independent of its canonical binding motif or ROS levels [49]. These findings suggest that NRF2 may also function as a transcriptional repressor via non-canonical mechanisms, which could help explain the observed downregulation of *SELENOP* in our study.

To validate the role of SeP in regulating intracellular selenium utilization, we generated SeP knockout HepG2 cells and examined the resulting changes in selenoprotein expression, intracellular selenium levels, and redox-related phenotypes. In the absence of SeP, intracellular selenium levels increased significantly, accompanied by enhanced expression of selenium-sensitive selenoproteins, notably GPx1 and GPx4. SeP is the second most abundant selenoprotein in the mouse liver at both the mRNA and protein levels [50], and uniquely contains 10 Sec residue, suggesting its central role in hepatic selenium utilization. Accordingly, suppression of SeP biosynthesis may redirect intracellular selenium toward the synthesis of other functional selenoproteins. These findings suggest that SeP functions not only as a transporter but also influences the intracellular availability of selenium for selenoprotein biosynthesis.

Furthermore, the regulatory impact of SeP suppression appears to be cell type dependent. In HuH7 cells, which have lower basal SeP expression than HepG2 cells, SeP knockdown did not significantly alter GPx4 protein levels (Supplementary Fig. 7). This variation may be partly explained by differences in baseline SeP expression, as confirmed by publicly available transcriptomic data from the Human Protein Atlas [51].

To further explore redox-related outcomes of SeP deficiency, we analyzed lipid hydroperoxide levels and ferroptosis sensitivity in SeP KO HepG2 cells. Consistent with enhanced antioxidant selenoprotein expression, SeP-deficient cells showed reduced lipid hydroperoxide accumulation and increased resistance to ferroptosis. These findings suggest that intracellular selenium retention following SeP downregulation reinforces redox defenses by promoting the synthesis of key selenoproteins such as GPx4.

In contrast, previous studies on copper overload have shown that inhibition of SeP secretion—without suppressing its biosynthesis—does not necessarily enhance antioxidant selenoprotein function; although hepatic selenium was retained, the protein levels of GPx and TrxR1 remained unchanged, while their enzymatic activities were reduced, likely due to selenium being sequestered in the intracellular SeP pool [52,53]. These findings underscore that downregulation of SeP biosynthesis, rather than inhibition of secretion alone, is critical for effective selenium redistribution.

Although our findings support the idea that selenium retained in SeP-deficient hepatocytes is redirected toward the synthesis of antioxidant selenoproteins, the precise fate of intracellular selenium remains unclear. It is unknown whether selenium or Sec-tRNA not used for SeP production is directly incorporated into other selenoproteins or diverted to alternative pathways. To clarify the functional consequences of this redistribution, it will be important to assess the enzymatic activities of GPx and TrxR1. Supporting this notion, SeP knockout in HepG2 cells conferred resistance to ferroptosis, indicating that the increased selenoprotein levels are not merely quantitative but also functionally active.

Our previous work in glioblastoma cells showed that SeP downregulation reduced selenoprotein levels and increased ferroptosis sensitivity—opposite to what we observed in HCC [54]. This difference may stem from cell-type–specific SeP uptake mechanisms. ApoER2 is abundantly present in neurons throughout various brain regions, enabling efficient reuptake of extracellular SeP and making them more reliant on circulating selenium [55,56]. In contrast, hepatocyte-derived cells such as HepG2 express low levels of ApoER2 and primarily function as selenium exporters [51]. Thus, SeP downregulation in HCC promotes intracellular selenium retention and enhances antioxidant capacity. Tissue-specific responses to SeP deficiency have also been observed in normal organs. For example, in SeP KO mice, GPx1 and GPx4 mRNA and protein levels are decreased in the brain and testis—where ApoER2-mediated SeP uptake is essential—but increased in the heart, where the regulatory mechanism remains unclear [57]. These findings suggest that the role of SeP in redox regulation and ferroptosis may differ between cancer types and could also vary across normal tissues, depending on selenium uptake mechanisms and cellular context.

To validate our in vitro findings and evaluate the physiological relevance of SeP-mediated selenium redistribution, we analyzed selenium metabolism in SeP KO mice. Under a standard CE-2 diet, SeP KO mice showed no significant changes in hepatic selenium content or selenoprotein expression compared to wild-type controls, contrasting with the clear selenium accumulation observed in SeP-deficient HepG2 cells. This discrepancy likely reflects the high selenium content of the standard diet, which exceeds the saturation threshold for selenoprotein synthesis [25]. CE-2–fed mice also exhibit serum selenium levels (250–500 μg/L) far higher than typical human levels (100–130 μg/L) [58,59], potentially masking the effects of SeP suppression. Indeed, serum SeP levels are also strongly affected by the selenium supply, which should be considered when interpreting SeP levels as a biomarker.

To address this, we used a selenium-deficient diet (0.003 mg/kg diet) and examined SeP KO mice after two weeks. Under these conditions, SeP KO mice showed increased hepatic selenium levels and GPx1 expression, indicating enhanced intracellular selenium retention and utilization when SeP-mediated export is absent. These in vivo results align with our cell-based findings and confirm that SeP suppression promotes selenium retention and antioxidant selenoprotein synthesis, but only under limited selenium supply.

Original studies characterizing SeP knockout mice demonstrated that selenium levels in the brain are markedly reduced under selenium-deficient conditions, highlighting the essential role of SeP in selenium delivery to the brain [25]. We previously extended these findings by showing that SeP-mediated selenium supply is critical for maintaining antioxidant selenoprotein expression in the brain, and that its deficiency accelerates the decline in brain selenium and increases oxidative stress and ferroptosis susceptibility during selenium deficiency [60]. This suggests that different organs rely on distinct selenium sources—dietary selenium versus SeP—for their selenium needs, and that selenium-dependent antioxidant defense may also vary by organ.

Recent studies have highlighted the potential of targeting oxidative stress regulation in cancer therapy, including inhibition of antioxidant selenoproteins such as *TXNRD1* and upstream regulators like NRF2 [9,61,62]. However, NRF2 is a key transcription factor that regulates a wide array of antioxidant and metabolic genes, and its global inhibition could cause undesirable off-target effects, raising concerns about the safety of directly targeting NRF2 in therapeutic setting. In this context, our findings suggest an alternative strategy: promoting SeP expression and secretion to modulate intracellular selenium availability. Rather than inhibiting antioxidant enzymes directly, enhancing SeP may reduce hepatic selenium retention and thereby limit the synthesis of selenoproteins such as GPx1 and GPx4, which support cancer cell survival under oxidative stress. This approach could provide a more selective way to influence redox balance in HCC.

Despite these promising findings, several limitations remain. While our study identifies NRF2 as a key modulator of selenium metabolism in HCC, the precise upstream regulatory mechanisms remain to be clarified, and other factors may also be involved. Furthermore, although SeP downregulation enhanced antioxidant capacity and ferroptosis resistance in vitro, it remains to be clarified whether similar adaptations occur in vivo, particularly within the complex tumor microenvironment. HCC induction in SeP knockout mice under selenium-deficient conditions could help clarify the causal relationships among NRF2 activation, SeP suppression, selenium redistribution, and ferroptosis resistance during liver tumor progression. However, such models require long-term carcinogen exposure and precise dietary control, as standard rodent diets contain excessive selenium that may mask relevant changes in selenium metabolism and selenoprotein expression. Given these challenges, in vivo validation and mechanistic dissection of this selenium regulatory axis remain important future directions.

## Conclusion

In this study, we demonstrated that NRF2 activation is associated with decreased expression of SeP, suggesting a potential regulatory link between NRF2 signaling and selenium transport in hepatocytes. This SeP suppression appears to promote intracellular selenium retention and the preferential synthesis of antioxidant selenoproteins such as GPx1, GPx4, and TrxR1, enhancing redox capacity and ferroptosis resistance in HCC cells.

These findings suggest a previously unrecognized form of NRF2-associated selenium metabolic remodeling that may support tumor cell adaptation to oxidative stress. Rather than being a passive consequence of tumor progression, SeP downregulation could represent an active adaptive strategy employed by cancer cells. Targeting this remodeling axis may provide new opportunities for redox-based therapeutic interventions in HCC.

## CRediT authorship contribution statement

**Kotoko Arisawa:** Writing – review & editing, Writing – original draft, Visualization, Validation, Methodology, Investigation, Funding acquisition, Formal analysis, Data curation, Conceptualization. **Moeka Natori:** Visualization, Methodology, Investigation. **Tetta Hiranuma:** Visualization, Methodology, Investigation, Formal analysis. **Misaki Shimizu:** Validation, Investigation. **Yuto Yamazaki:** Investigation. **Yasuhiro Miki:** Investigation. **Takashi Toyama:** Writing – review & editing, Funding acquisition, Conceptualization. **Yoshiro Saito:** Supervision, Resources, Project administration, Funding acquisition, Conceptualization.

## Availability of data and materials

The authors declare that all data are available within the article under reasonable requirements. The materials e.g., SeP antibodies are also available on request.

## Declaration of generative AI and AI-assisted technologies in the writing process

During the preparation of this work the authors used ChatGPT by OpenAI in order to improve the English language and readability of the manuscript. After using this tool, the authors reviewed and edited the content as needed and takes full responsibility for the content of the publication.

## Funding

This work was supported in part by the Japan Society for the Promotion of Science (JSPS) Grants-in-Aid for Scientific Research (KAKENHI) [Grant Numbers 25292078, 20H00488, 20H05491, 21K19321, and 21H05270 for Y.S.; 22H04796 and 24K20660 for K.A.]. K.A. was also supported by a Young Investigator Grant from the Japan Society of Nutrition and Food Science.

## Declaration of competing interest

The authors declare that they have no known competing financial interests or personal relationships that could have appeared to influence the work reported in this paper.

## Data Availability

Data will be made available on request.
