# Supplementary material for: NRF2-dependent suppression of selenoprotein P expression promotes intracellular selenium metabolic remodeling and upregulation of antioxidant selenoproteins in hepatocellular carcinoma
Source: Redox Biol. 2025 Aug 13;86:103821. doi: 10.1016/j.redox.2025.103821 (PMC12391428; doi:10.1016/j.redox.2025.103821)
Supplement: Multimedia component 1 [file mmc1.pdf]

Supplementary table 1

Gene	Species	Forward Primer (5'-3')	Reverse Primer (5'-3')
GPX1	Human	CAGTCGGTGTATGCCTTCTCG	GAGGGACGCCACATTCTCG
GPX1	Mouse	AGTCCACCGTGTATGCCTTCT	GAGACGCGACATTCTCAATGA
GPX4	Human	GAGGCAAGACCGAAGTAAACTAC	CCGAACTGGTTACACGGGAA
GPX4	Mouse	GCCTGGATAAGTACAGGGGTT	CATGCAGATCGACTAGCTGAG
SELENOP	Human	CCCCCAGCCTGGAGCATAAG	TGCACAGGTATCAGCTGGCTT
SELENOP	Mouse	AGCTCTGCTTGTTACAAAGCC	GAGGGACGCCACATTCTCG
TXNRD1	Human	ATATGGCAAGAAGGTGATGGTCC	GGGCTTGTCCTAACAAAGCTG
TXNRD1	Mouse	CCCACTTGCCCCAACTGTT	GGGAGTGTCTTGGAGGGAC
KEAP1	Human	CTGGAGGATCATACCAAGCAGG	GAACATGGCCTTGAAGACAGG
HO-1	Human	CCAGCAACAAAGTGCAAGATTC	TCACATGGCATAAAGCCCTACAG
GAPDH	Human	GCACCGTCAAGGCTGAGAAC	TGGTGAAGACGCCAGTGGA
GAPDH	Mouse	AACTTTGGCATTGTGGAAGG	ACACATTGGGGGTAGGAACA

Supplementary table 1. The qPCR primers used in this study

Supplementary table 2

Parameter	Setting
**RF power**	1550 W
**Plasma gas flow rate**	15.0 L/min
**Auxiliary gas flow rate**	0.90 L/min
**Nebulizer gas flow rate**	1.03 L/min
**Nebulizer type**	Micromist
**Peristaltic pump speed**	0.1 revolutions per second (rps)
**Spray chamber temperature**	2 °C
**Sampler/skimmer cone material**	Nickel
**Ion lens system**	x-Lens
**Scanning mode**	Peak hopping
**Peak pattern**	1 point
**Number of replicates**	3
**Sweeps per replicate**	10
**Collision/reaction cell mode**	Oxygen (O_2) mode
**O_2 gas flow rate**	30%

Supplementary table 2. Instrumental settings for ICP-MS-based elemental analysis

Supplementary table 3

Parameter	Details
**Standard curve**	$y = 6.025 \times 10^2 x + 2.333$
**Correlation coefficient (R^2)**	0.9999
**Certified reference material**	Seronorm™ Trace Elements Serum L-2 RUO
**Lot number**	1801803
**Certified Se value**	139 ng/mL
**95% Confidence interval**	111–167 ng/mL
**Measured average (n = 3)**	150 ng/mL
**Intra-day precision (RSD%)**	5.7%
**Inter-day precision (RSD%)**	10.5%

Supplementary table 3. Validation of selenium (Se) measurement by ICP-MS

Supplementary table 4

GeneID	GeneName	p_value	Tumor_mean	Normal_mean
ENSG00000211450	SELENOH	**2.57E-09**	17.19	9.84
ENSG00000131871	SELENOS	**3.24E-06**	52.52	34.70
ENSG00000198431	TXNRD1	**1.52E-05**	7.79	4.25
ENSG00000179918	SEPHS2	**5.80E-05**	1221.39	944.02
ENSG00000162430	SELENON	**0.0001**	13.05	8.87
ENSG00000113811	SELENOK	**0.0002**	32.43	25.87
ENSG00000183291	SELENOF	**0.0012**	12.06	9.71
ENSG00000167468	GPX4	**0.0013**	740.87	599.76
ENSG00000176153	GPX2	**0.0022**	576.73	323.05
ENSG00000233276	GPX1	**0.0035**	1496.64	1161.13
ENSG00000198843	SELENOT	**0.0043**	12.57	10.46
ENSG00000198832	SELENOM	**0.0074**	15.08	8.91
ENSG00000184470	TXNRD2	**0.0122**	5.40	4.61
ENSG00000197763	TXNRD3	**0.0156**	0.63	0.52
ENSG00000073169	SELENOO	0.0510	18.96	15.91
ENSG00000211448	DIO2	0.1009	0.11	0.08
ENSG00000211445	GPX3	0.1699	487.46	462.18
ENSG00000138018	SELENOI	0.2463	3.36	3.24
ENSG00000197406	DIO3	0.2843	1.99	1.32
ENSG00000250722	SELENOP	0.3186	23.00	22.50
ENSG00000186838	SELENOV	0.3206	0.0062	0.0031
ENSG00000198736	MSRB1	0.5686	135.39	134.69
ENSG00000178980	SELENOW	0.6527	69.22	50.90
ENSG00000211452	DIO1	0.8097	28.85	23.30
ENSG00000198704	GPX6	0.8692	0.0008	0.0002

Supplementary table 4. Summary of selenoprotein gene expression differences between tumor and normal liver tissues in TCGA-LIHC. Each row represents a selenoprotein gene, showing the corresponding Ensembl Gene ID, gene name, average expression in primary tumor and normal liver tissue samples (TPM), and the p-value obtained from the Wilcoxon rank-sum test. Statistically significant differences ($p < 0.05$) are highlighted in bold.

Supplementary table 5

GeneID	GeneName	p_value	Tumor_mean	Normal_mean
ENSG00000211450	SELENOH	**1.51E-10**	14.24	7.26
ENSG00000131871	SELENOS	**6.75E-09**	50.58	24.95
ENSG00000113811	SELENOK	**7.98E-09**	32.46	19.09
ENSG00000233276	GPX1	**1.54E-08**	1637.84	779.07
ENSG00000167468	GPX4	**2.17E-08**	830.69	425.73
ENSG00000198843	SELENOT	**1.45E-07**	13.51	8.15
ENSG00000162430	SELENON	**2.75E-07**	13.05	5.00
ENSG00000197763	TXNRD3	**5.27E-07**	0.70	0.43
ENSG00000198832	SELENOM	**1.06E-05**	17.07	2.79
ENSG00000179918	SEPHS2	**1.62E-05**	1215.33	750.85
ENSG00000183291	SELENOF	**2.96E-05**	13.74	7.38
ENSG00000198736	MSRB1	**0.0001**	153.97	107.90
ENSG00000198431	TXNRD1	**0.0004**	10.81	1.78
ENSG00000138018	SELENOI	**0.0007**	4.13	2.58
ENSG00000178980	SELENOW	**0.0027**	66.00	43.55
ENSG00000176153	GPX2	**0.0036**	612.25	218.55
ENSG00000184470	TXNRD2	**0.0046**	5.87	4.20
ENSG00000073169	SELENOO	**0.0046**	19.18	15.42
ENSG00000211445	GPX3	**0.0074**	599.81	306.74
ENSG00000211448	DIO2	**0.0079**	0.09	0.00
ENSG00000198704	GPX6	**0.0145**	0.001	0.000
ENSG00000250722	SELENOP	0.1861	25.72	22.12
ENSG00000211452	DIO1	0.4786	28.94	26.14
ENSG00000186838	SELENOV	0.7496	0.002	0.003
ENSG00000197406	DIO3	0.9053	1.29	1.34

Supplementary table 5. Summary of selenoprotein gene expression differences between paired tumor and normal liver tissue samples in TCGA-LIHC. Each row represents a selenoprotein gene, showing the corresponding Ensembl Gene ID, gene name, average expression in primary tumor and matched normal tissue samples (TPM), and the p-value obtained from a paired t-test. Statistically significant differences ($p < 0.05$) are highlighted in bold.

Supplementary Figure 1

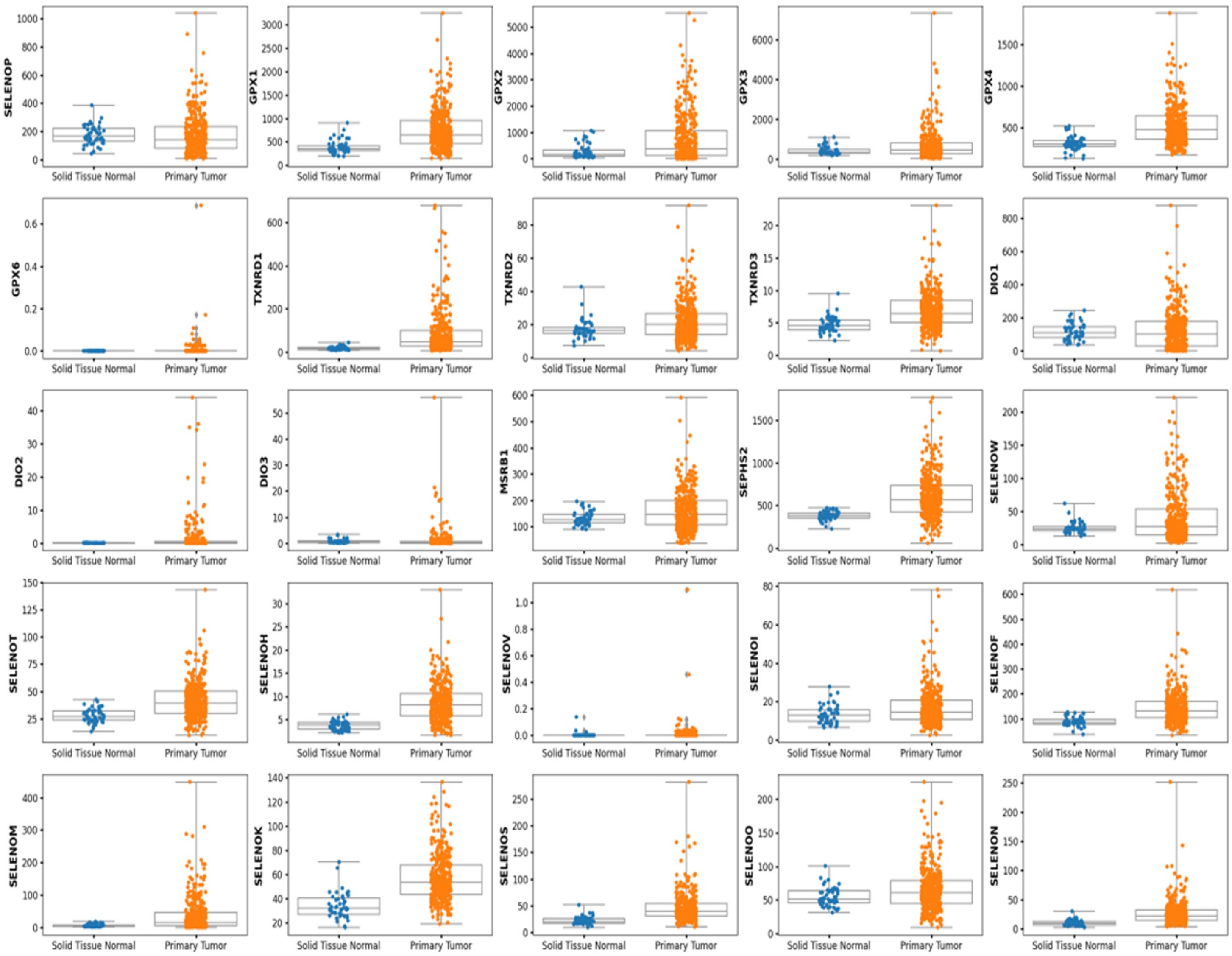

Supplementary Figure 1. Expression of all 25 human selenoprotein genes in TCGA-LIHC tumor and normal tissues.

Box plots showing TPM values of all 25 selenoprotein genes in tumor (n = 371) and normal liver tissues (n = 50) from the TCGA-LIHC dataset. Data correspond to the analysis presented in Figure 1A. Genes are shown in a fixed order for comparison, and p-values were calculated using the Wilcoxon rank-sum test.

Supplementary Figure 2

A Patient with **lower *SELENOP* expression** in tumor tissue (compared to paired normal tissue)

SELENOP

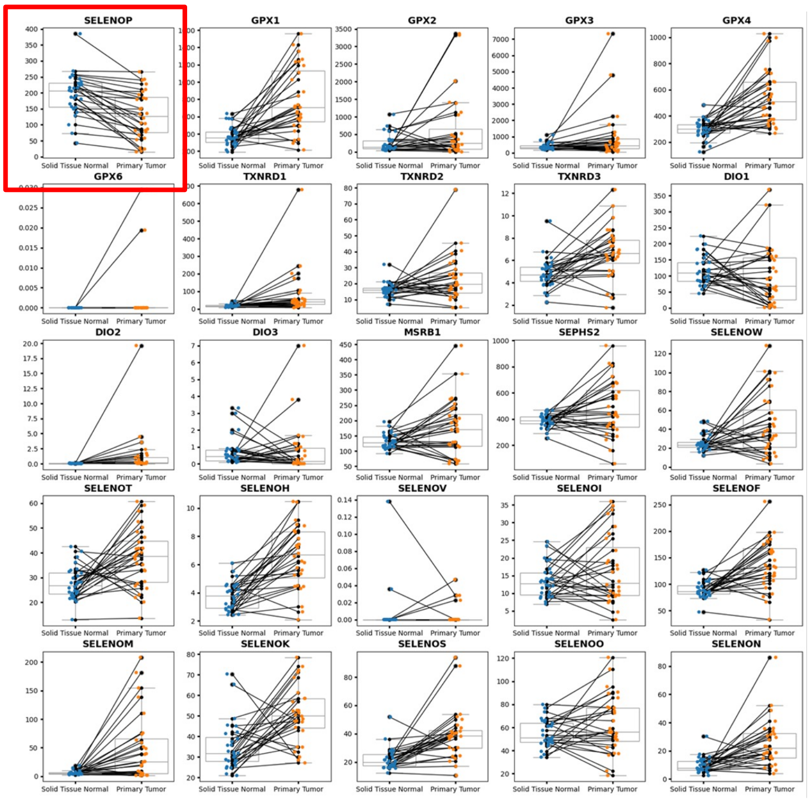

B Patient with **higher *SELENOP* expression** in tumor tissue (compared to paired normal tissue)

SELENOP

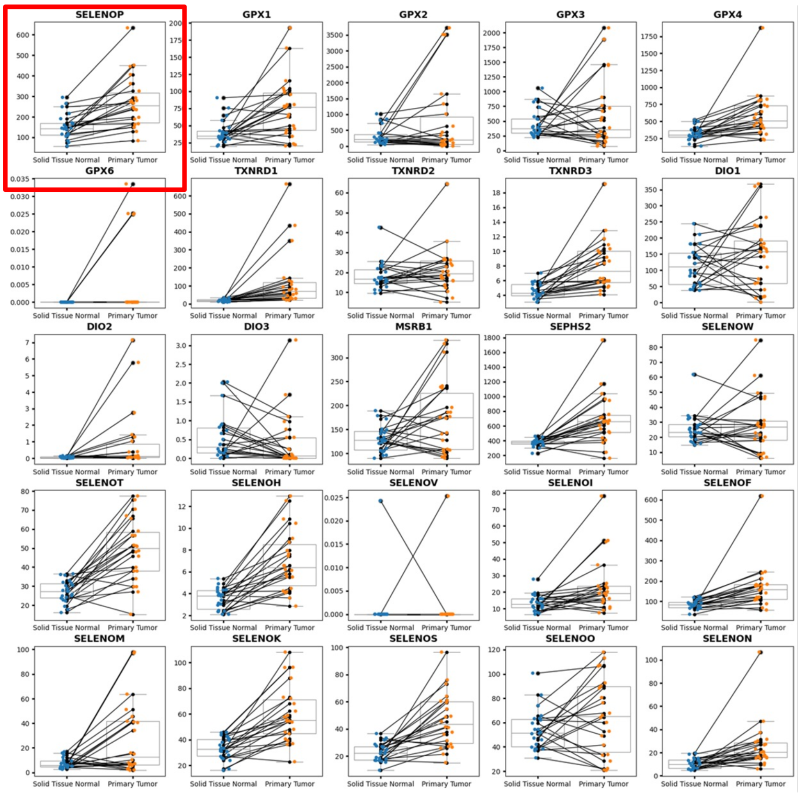

Supplementary Figure 2. Paired analysis of all 25 human selenoprotein genes in TCGA-LIHC samples.
Box plots showing TPM values of 25 selenoprotein genes in tumor and matched adjacent normal tissues from 50 HCC patients in the TCGA-LIHC dataset. Data are stratified into two subgroups based on SELENOP expression pattern in tumor tissues relative to matched normal tissues: decreased (upper) and increased (lower), as shown in Figure 1C. Genes are displayed in a consistent order across both subgroups.

Supplementary Figure 3

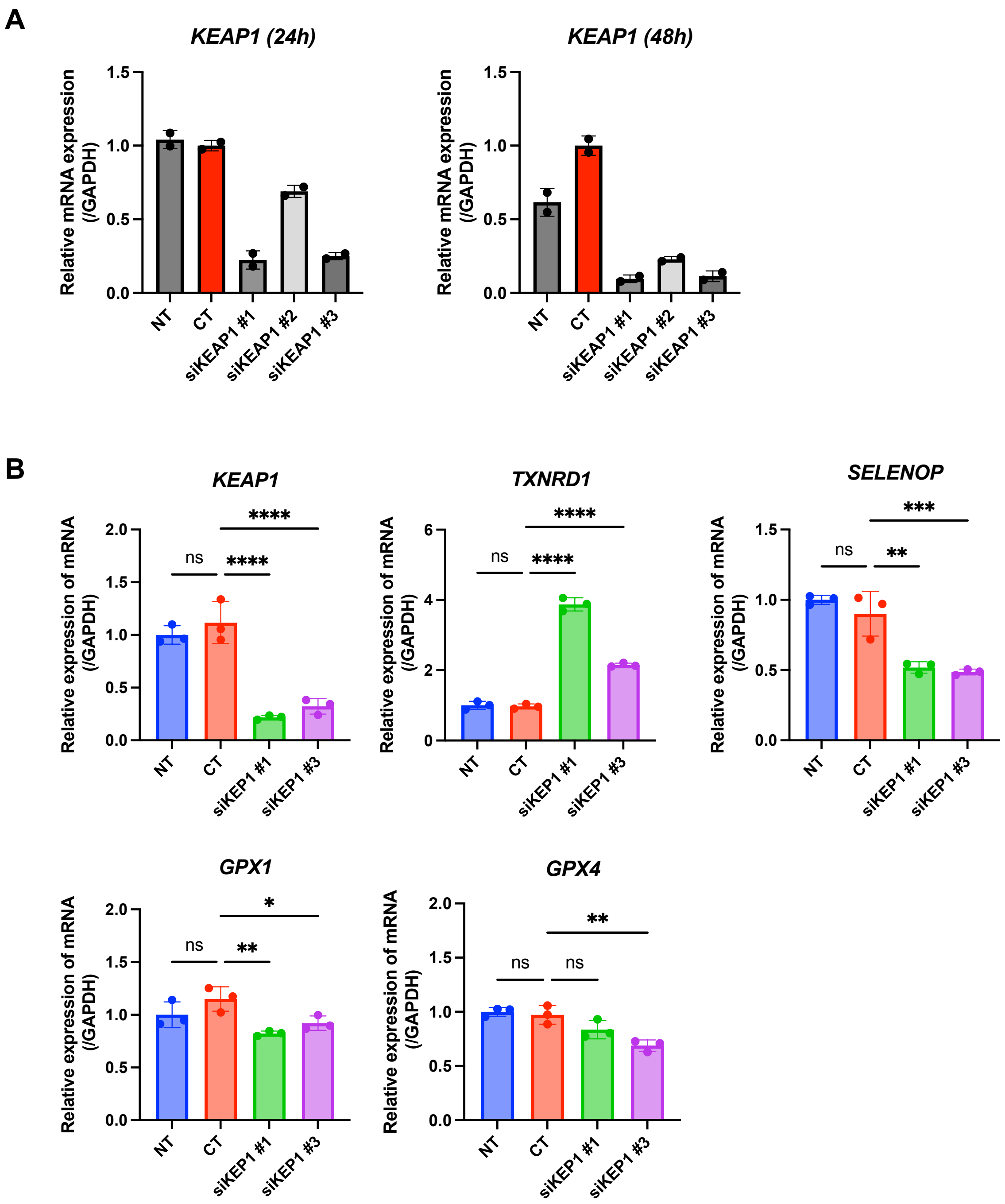

Supplementary Figure 3. Effects of KEAP1 knockdown on selenoprotein expression in HepG2 cells.

(A) Knockdown efficiency of three different KEAP1 siRNA sequences. HepG2 cells were transfected with 10 nM of each KEAP1 siRNA using a transfection reagent and cultured for 48 hours. KEAP1 mRNA expression was quantified by RT-qPCR and normalized to GAPDH. “NT” indicates non-treated cells; “CT” refers to control siRNA-transfected cells (negative control). n = 2 per group. Among the three siRNAs, siRNA #2 showed relatively weak knockdown efficiency and was excluded from subsequent experiments. siRNA #1 and #3 were used in all following analyses.

(B) mRNA expression levels of selected selenoproteins following KEAP1 knockdown in HepG2 cells. Data are presented as mean ± SD (n = 3). Statistical significance: *$p < 0.05$, **< 0.01, ***< 0.001, **** < 0.0001 vs. CT (control siRNA group), analyzed by Dunnett’s test.

Supplementary Figure 4

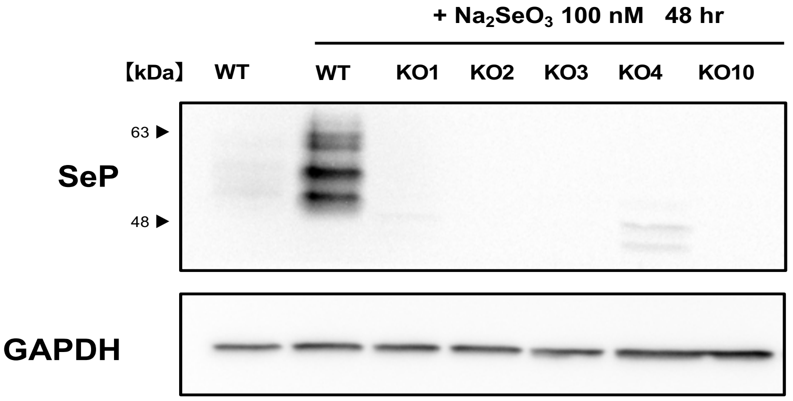

Supplementary Figure 4 Establishment of SeP knockout (SeP KO) cells using the CRISPR/Cas9 system.
HepG2 cells were transfected with crRNA, tracrRNA, and Cas9 plasmid to knockout the SeP gene. Cloned cells were cultured in medium containing 100 nM sodium selenite for 48 hours, and disruption of the SeP gene was confirmed by Western blotting.

Supplementary Figure 5

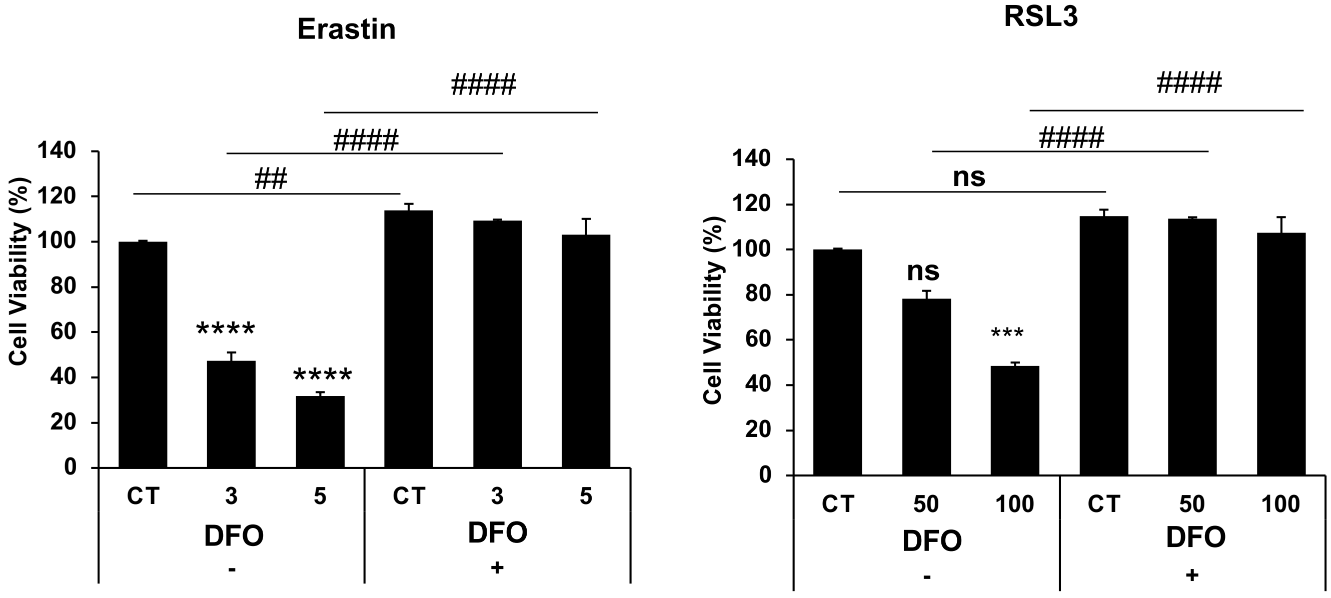

Supplementary Figure 5 Determination of ferroptosis-inducing conditions in wild-type HepG2 cells.

HepG2 cells were treated with 3 μM or 5 μM erastin, or 50 nM or 100 nM RSL3 for 24 hours. Cell viability was assessed to determine the concentrations that induced approximately 50% cell death. Co-treatment with 10 μM deferoxamine (DFO), an iron chelator, completely rescued cell death under all conditions, confirming that the observed cytotoxicity was ferroptotic in nature.

Data are presented as mean ± SD (n = 3). Statistical significance: *p < 0.05, **p < 0.01, ***p < 0.001, ****p < 0.0001 vs. CT; #p < 0.05, ##p < 0.01, ###p < 0.001, ####p < 0.0001 for 0 μM DFO vs. 10 μM DFO (Tukey’s multiple comparison test).

Supplementary Figure 6

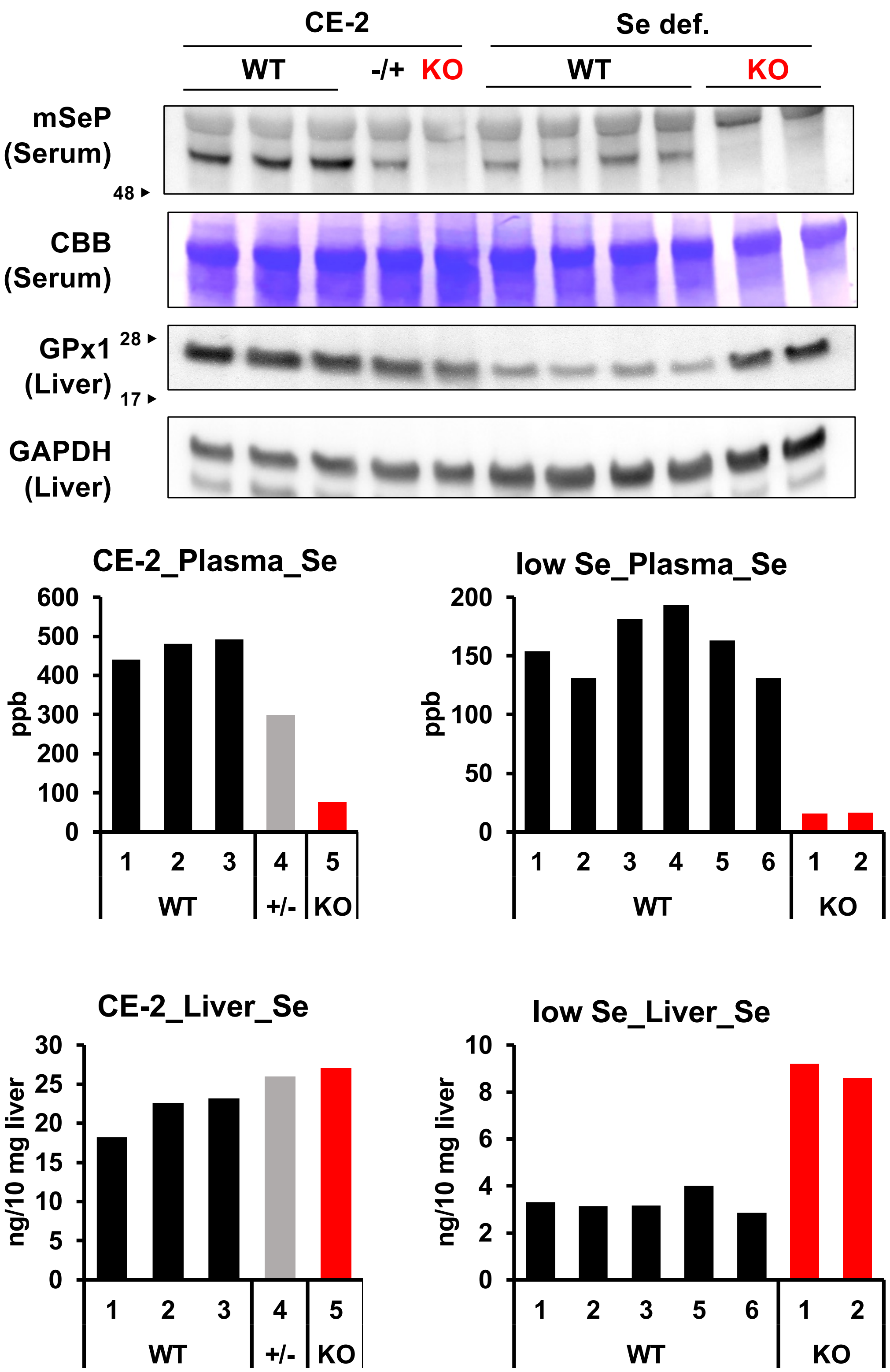

Supplementary Figure 6 Determination of ferroptosis-inducing conditions in wild-type HepG2 cells.
HepG2 cells were treated with 3 μM or 5 μM erastin, or 50 nM or 100 nM RSL3 for 24 hours. Cell viability was assessed to determine the concentrations that induced approximately 50% cell death. Co-treatment with 10 μM deferoxamine (DFO), an iron chelator, completely rescued cell death under all conditions, confirming that the observed cytotoxicity was ferroptotic in nature.
Data are presented as mean ± SD (n = 3). Statistical significance: *$p < 0.05$, **$p < 0.01$, ***$p < 0.001$, [illegible]

Supplementary Figure 7

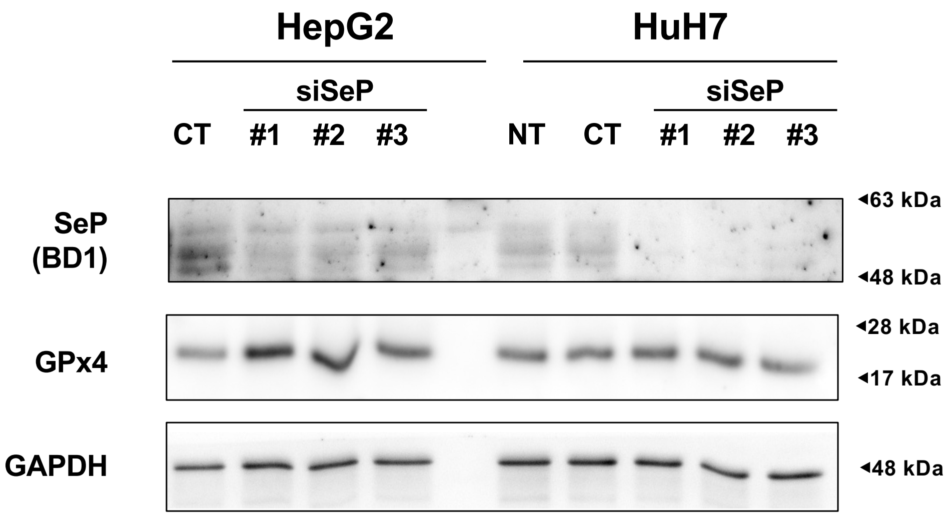

Supplementary Figure 7 Effects of SeP knockdown on GPX4 expression in HepG2 and HuH7 cells.
HepG2 and HuH7 cells were transfected with three different SeP-targeting siRNAs and cultured for 48 hours. SeP and GPX4 protein expression was evaluated by western blotting. In HepG2 cells, SeP knockdown increased GPX4 expression, while in HuH7 cells—where basal SeP expression is relatively low—GPX4 levels remained unchanged.
